# The Current State-of-the-Art Identification of Unknown Proteins Using Mass Spectrometry Exemplified on De Novo Sequencing of a Venom Protease from *Bothrops moojeni*

**DOI:** 10.3390/molecules27154976

**Published:** 2022-08-05

**Authors:** Simone König, Wolfgang M. J. Obermann, Johannes A. Eble

**Affiliations:** 1 IZKF Core Unit Proteomics, Interdisciplinary Center for Clinical Research, University of Münster, Röntgenstr. 21, 48149 Münster, Germany; 2 Institute of Physiological Chemistry and Pathobiochemistry, University of Münster, Waldeyer-Str. 15, 48149 Münster, Germany

**Keywords:** snake venom, gas phase peptide ion fragmentation, mass spectrometry, spectrum quality, MS/MS, CID

## Abstract

(1) Background: The amino acid sequence elucidation of peptides from the gas phase fragmentation mass spectra, de novo sequencing, is a valuable method for the identification of unknown proteins complementary to Edman sequencing. It is increasingly used in shot-gun mass spectrometry (MS)-based proteomics experiments. We review the current state-of-the-art and use the identification of an unknown snake venom protein targeting the human tissue factor (TF) as an example to describe the analysis process based on manual spectrum interrogation. (2) Methods: The immobilized TF was incubated with a crude *B. moojeni* venom solution. The potential binding partners were eluted and further purified by gel electrophoresis. Edman degradation was performed to elucidate the N-terminus of the 31 kDa protein of interest. High-resolution MS with collision-induced dissociation was employed to generate peptide fragmentation spectra. Sequence tags were deduced and used for searches in the NCBI and Uniprot databases. Protein matches from the snake species were further validated by target MS/MS. (3) Results: Sequence tag D [K/Q] D [I/L] VDD [K/Q] led to a snake venom serine protease (SVSP) from lancehead *B. jararaca* (P81824). With target MS/MS, 24% of the SVSP sequence were confirmed; an additional 41% were tentatively assigned by data-independent MS. Edman sequencing provided information for 10 N-terminal amino acid residues, also confirming the match to SVSP. (4) Conclusions: The identification of unknown proteins continues to be a challenge despite major advances in MS instrumentation and bioinformatic tools. The main requirement is the generation of meaningful, high-quality MS peptide fragmentation spectra. These are used to elucidate sufficiently long sequence tags, which can subsequently be submitted to searches in protein databases. This basic method does not require extensive bioinformatics because peptide MS/MS spectra, especially of doubly-charged ions, can be analysed manually. We demonstrated the procedure with the elucidation of SVSP. While de novo sequencing quickly indicates the correct protein group, the validation of the entire protein sequence of amino acid-by-amino acid will take time. Reasons are the need to properly assign isobaric amino acid residues and modifications. With the ongoing efforts in genomics and transcriptomics and the availability of ever more data in public databases, the need for de novo MS sequencing will decrease. Still, not every animal and plant species will be sequenced, so the combination of MS and Edman sequencing will continue to be of importance for the identification of unknown proteins.

## 1. Mass Spectrometry-Based De Novo Sequencing

**Figure 1:**
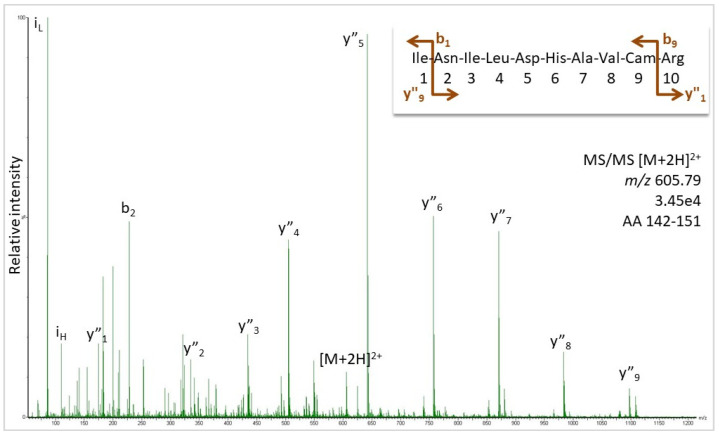
MS/MS spectrum of the doubly-charged peptide ion (*m/z* 605.79) detected in the tryptic digest of a 31 kDa protein isolated from *B. moojeni*. Peaks were labelled as b- and y-ions for sequence INILDHAVCamR of peptide 142–155 from the protein with the accession number NCBI AAB34465.1, as calculated in Supplementary Figure S1. For the original spectrum, also see Figure S1.

Mass spectrometry (MS) has complemented the Edman sequencing of peptides and proteins for many years with regard to sequence determination and even replaced it for many applications. Its advantages are the comparatively little sample consumption, high mass accuracy, and the capability to fragment peptides in the gas phase within the mass spectrometer. The latter process delivers ion signals for individual amino acid (AA) residue losses allowing sequence elucidation based on accurate fragment ion mass information (for an example, see Figure 1). Furthermore, MS is not hampered by blocked peptide termini or otherwise modified AAs. An excellent review of the analytical method employed for the purpose, namely tandem MS, was published in 2010 and is still the best resource to understand the basic technology behind collision cell MS/MS [1]. Moreover, the relation between the peptide structure and observed fragment ion series is discussed there, as well as the extraction of sequence information from the MS/MS spectra of protonated peptide ions.

Spectra of high quality, as shown in Figure 1, are required for a confident sequence assignment. Although obvious, this fact needs to be underlined because, increasingly, automatic measurement and data analysis routines are employed for the analysis of hundreds and thousands of proteins in one run. Not all of the spectra in such a big data set are of sufficient quality and the information content to allow a reliable sequence assignment or de novo sequencing. For an introduction to the essential features of a peptide MS/MS spectrum for laymen applicants of the technology, see reference [2].

Bioinformatics for data mining developed alongside the MS instrumentation and basic spectrum analysis tools are typically supplied with the instrument. Simple programs for de novo analysis mimic manual calculation and suggest sequence tags, namely stretches of ~4–8 AA, based on a clearly distinguishable ion series (for an example, see Supplementary Figure S2). More advanced software tools provide (semi-)automatic routines. The performance of five algorithms was evaluated in the year 2006 and found to strongly depend on—not surprisingly—spectral quality [3]. Furthermore, a dependence on the data source, namely the spectrometer type, was noted. Since then, more tools have been programmed. Some try to accommodate the results from different fragmentation methods (collision-induced dissociation (CID), electron transfer dissociation (ETD), and higher-energy collisional dissociation (HCD)) [4,5], while others improve the analyses from certain instrument types [6] or focus on ultrahigh-resolution data [7]. Generally, the challenge of the identification of a peptide from its spectrum alone is recognized even for state-of-the-art algorithmic approaches [8]. Complicated trainable mathematical models (e.g., Lagrangian relaxation [9]) were developed to tackle problems with respect to run time and the accuracy and flexibility concerning ion types. Increasingly, deep learning [10,11] and neural networks [12] are implemented. Despite all these efforts, the main problem remains the need to generate spectra of sufficient fragment ion intensity so that software has a chance to extract meaningful information.

In addition, for the identification of proteins, it does not suffice to collect a number of sequence tags because they still need to be aligned in the correct order. For this purpose, a recent paper proposes the use of multiple unspecific hydrolysis methods in combination with a contigscaffolding strategy, which was inspired by genome assembly techniques [13]. This paper also serves as a review of earlier efforts in that regard.

**Figure 2:**
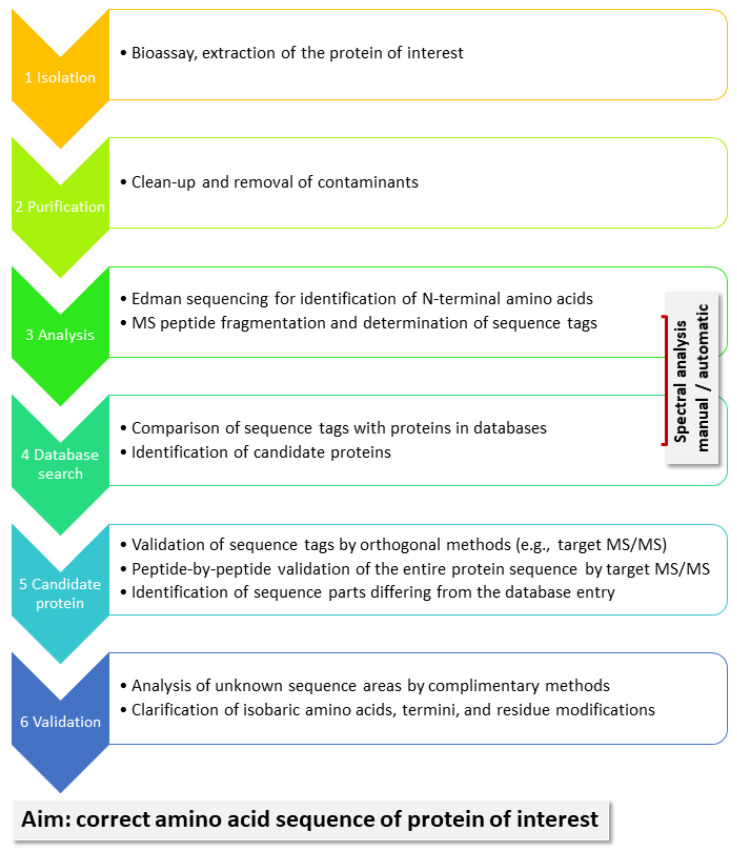
General workflow for the identification of unknown proteins of interest. (**1**) They are typically isolated based on measurable bioactivity and (**2**) subsequently purified using suitable methods such as chromatography and gel electrophoresis. (**3**) The clean analyte is then subjected to chemical analysis. Edman sequencing delivers information for maximal 50 N-terminal AAs, typically of 10–15. MS-based sequencing generates sequence information by gas phase fragmentation of enzymatically produced peptides. (**4**) Both methods produce pieces of the sequence (tags), which are compared to protein databases. If these tags are sufficiently long and characteristic, protein matches can be detected, which serves as hypothesis for (**5**) validation. Because the protein sequence from the database may be homologous but not completely identical to that of the analyte, target analyses need to determine the differences. At this point, sufficient experimental evidence may be available to confidently assign the analyte to a certain protein group, which allows the biologists to continue their studies. (**6**) However, the entire protein sequence still has to be determined AA-for-AA to fill in the missing positions. This process can be time-consuming depending on the available analytical technology and the structure of the protein. For instance, highly glycosylated or otherwise modified proteins may complicate the analysis.

A general workflow for the identification of unknown proteins is shown in Figure 2. Proteins are of interest because they present with certain bioactivity, as we demonstrate in our example below. They need to be isolated from their biological matrix and purified before they can be subjected to chemical analysis. Edman sequencing has the advantage of delivering the N-terminal AAs, typically 10–15. MS-based sequencing, in contrast, generates sequence information for enzymatically, typically, produced peptides. Depending on the protein sequence, in particular, the presence of suitable cleavage sites, as well as factors such as peptide size and ionization efficiency, not all peptides may be equally well detected. Especially finding the termini could be a challenge. In order to assemble sequence tags, which are sufficiently long (6–10 AA) to deliver meaningful information in database searches, the fragment ion spectra of peptides in the mass range from 1000 to 2500 Da tend to be useful. They present two and possibly three charge states, which can be handled in manual spectral analysis. Good spectra may also be generated from larger peptides, but they may charge up higher and are more easily analyzed with the help of deconvolution software. One specific sequence tag may suffice to find a candidate protein in a protein database, such as Uniprot; it needs to be subsequently validated by target analysis. Because the protein sequence from the database will likely be homologous but not completely identical to that of the analyte, MS/MS will confirm some, but not all of the possible peptides. Still, sufficient experimental evidence may be available to confidently assign the analyte to a certain protein group and allow the biologists to proceed with their studies. However, if the entire protein sequence of the analyte is needed, it has to be determined AA-for-AA to fill in the missing positions. This process can be time-consuming depending on the available analytical technology and the structure of the protein. For instance, highly glycosylated or otherwise modified proteins may complicate the analysis.

If de novo sequencing needs to be performed on a routine basis based on shot-gun analysis, the help of a bioinformatician should be secured to deal with the increasingly challenging software tools. However, there is no reason to shy away from manually tackling the occasional sequencing problem. Resources for the training of spectrum interpretation are abundantly available in the scientific literature [2,14,15,16,17] and on the internet. Manual spectrum control is also an important sanity check of the output of automatic analysis routines.

Exemplarily, we present here the identification of an unknown 31 kDa protein from the venom of the lancehead *Bothrops moojeni* with high-resolution MS coupled to nanoflow reversed-phase liquid chromatography (LC) using no sequencing software tools at all.

## 2. Analysis of *B. moojeni* Venom Protein

The protein of interest was isolated in a screen for venom components that target the human tissue factor (TF) across different *Bothrops* species. The TF starts the blood coagulation cascade that triggers the conversion of fibrinogen to fibrin. In cancer patients, fibrin forms cohesive bridges between the platelets and tumor cells that support an aberrant interplay between the two cell types. This leads to the formation of tumor cell–platelet aggregates that cause cancer-associated thrombosis and hematogenous metastasis, finally increasing the death risk of cancer patients. Venom components that target the TF might interfere with the blood coagulation cascade at this step and thereby prevent cancer-associated thrombosis and hematogenous metastasis [18].

We conducted our experiments with the hope that our protein had similarities to known sequences. That being the case, comparisons of experimentally obtained sequence tags with databases could provide hints on the unknown protein. The elucidation of short sequence stretches would lead to a protein sequence hypothesis without having to use an extensive multi-enzyme procedure, as discussed in reference [13].

**Figure 3:**
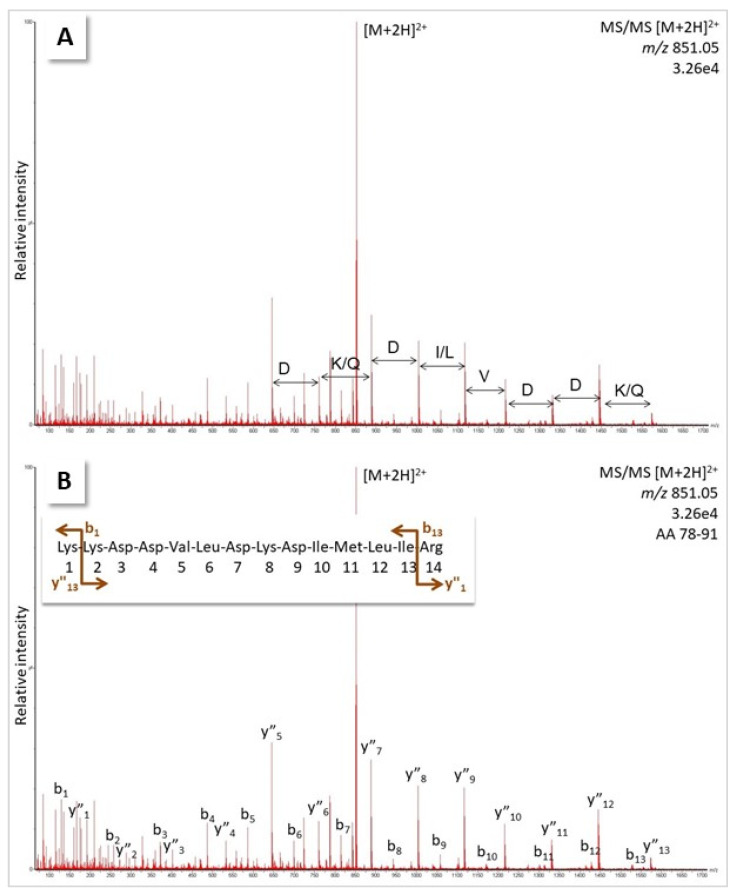
MS/MS spectrum of a doubly-charged peptide ion (*m/z* 851.05) detected in the tryptic digest of a 31 kDa protein isolated from *B. moojeni*. (**A**) Differences between peaks of a potential ion series were calculated and tentatively assigned to AA residues. (**B**) Ion series assigned for sequence KKDVLDKDIMLIR (AA 78–91, AAB34465.1) from lancehead *B. jararaca*. For the original spectrum, see Supplementary Figure S5; for calculation of expected ions, see Supplementary Figure S6.

**Figure 4:**
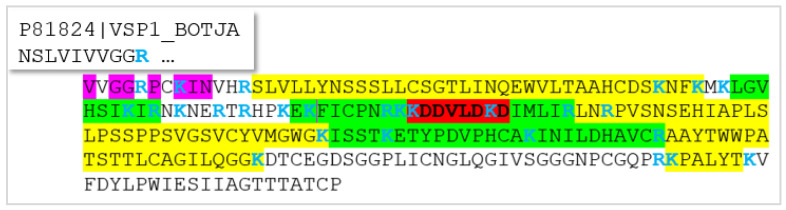
The protein sequence of the platelet-aggregating enzyme from *B. jararaca* (NCBI AAB34465.1). In Uniprot, the same sequence was detected, which contained an additional ER-targeting propeptide at the N-terminus (P81824). The sequence tag, which led to the discovery of this protein, is marked in red and bold. Trypsin cleavage sites K and R are blue. Sequence parts, which were validated by target MS/MS, are colored in green, with those assigned by DIA in yellow. For the uncolored areas, no spectral evidence could be generated, although it was attempted. The AA residues in purple were confirmed by Edman sequencing, which suggested the N-terminus VIGG [R/L] P [X/C] KIN with two sites remaining unclear.

A first attempt at generating sequence tags from a 1D-gel band of the 31 kDa protein from *B. moojeni*, isolated by affinity capturing using the TF as bait, was not successful because no spectral, respective sequence tag information could be generated, which led to any snake protein when tested against the NCBI and Uniprot databases. Rather, tags were produced that were too short and unspecific or matched sequences from bacteria. We thus decided to add another purification step, namely 2D-mini gel electrophoresis, using a pH 3–10 p*I*-strip. A smear across the entire gel was detected, ending in a spot at ~pH 10, which was excised and further analysed by tryptic digestion and MS/MS (Supplementary Figure S3). Not surprisingly, some high-quality spectra obtained from that digest led to the identification of trypsin (peptide VATVSLPR, fragment -VLEGNEQ-; Supplementary Figure S4), the enzyme added to the sample for protein cleavage during the sample preparation. Eventually, an eight AA-long tag (D [K/Q] D [I/L] VDD [K/Q]) was assigned in a spectrum of a doubly-charged peptide at *m*/*z* 851.05 by calculating the difference between the major peaks of the ion series at *m*/*z* values higher than the parent ion (Figure 3A). Thereby, it is important to know that isoleucine and leucine residues have the exact same mass and cannot be distinguished by MS analysis alone. Lysine and glutamine are very close in mass and were also not differentiated at this point. Moreover, it was not clear if the tag had to be read forward or backward. When checking it in reverse order (input: [K/Q] DDV [IL] D [KQ] D) against the NCBI database using the Protein Prospector pattern search, several snake venom proteases were returned, and the serine protease, AAB34465.1 from another lancehead, *B. jararaca*, was selected as the tentative hypothesis (Figure 4). In Uniprot, the same sequence was detected with an additional ER-targeting propeptide at the N-terminus (P81824). This spectrum, thus, potentially corresponded to the tryptic peptide KKDDVLDKDIMLIR (AA 78–91), and that match was confirmed (Figure 3B). It showed a complete b- and y-ion series, which instilled high confidence in the assignment.

Subsequently, target MS/MS experiments for all the tryptic peptides of this protein sequence were conducted, in addition to data-independent MS analysis (DIA), using the *B. jararaca* protein sequences as the database. Figure 1 shows the MS/MS for the peptide at *m/z* 605.79, where the peaks matched the expected ions for sequence INILDHAVCamR (Cam: carbamidomethylated cysteine). Further MS/MS spectra are supplied in Supplementary Figures S7–S14, which confirm the assignment of sequence regions AA 51–59, 70–77, 79–91, 126–141, and 142–151 as visualized by the color-coding in Figure 4. For the other peptides, target analysis, although attempted, was not successful. The DIA additionally suggested the presence of AA 1–8 (score 6.03), 14–48 (score 5.68), 92–125 (score 6.45), 152–173 (score 6.18), and 205–211 (score 6.59) (Supplementary Excel File), but this information has to be noted cautiously because assignment scores below 7 indicate weak to questionable matches of spectral data. For comparison, all the data, which were confirmed by target MS/MS, showed scores from 7 to 9. Still, the DIA results should not be entirely discarded because MS and automatic analysis are quite useful for the sequencing of larger peptides presenting with higher charge states than two that make manual analysis cumbersome. We treat it as supportive evidence, which was indeed not confirmed for the N-terminus. While the DIA assigned the *B. jararaca* peptide VVGGRPCamK (based on only two fragment ions), Edman sequencing detected isoleucine in position 2. R_5_ and C_7_ were unclear in the Edman experiments, suggesting the terminal sequence VIGG [R/L] P [X/C] KIN. The presence of R or L in position 5 was tested by the target MS/MS, assuming C_7_ to be correct, but both AAs could not be confirmed by spectral evidence. The same was true for the C-terminus and a longer sequence stretch with no tryptic cleavage sites (the AA 174–204). Besides the size of the peptide, differing AAs in some positions compared to the *B. jararaca* protein and post-translational modifications can be responsible for the failure of peptide detection. For N_25_ and S_28_ (P81824, Figure 4), for instance, N- and O-linked glycosylation, respectively, were published [19].

**Figure 5:**
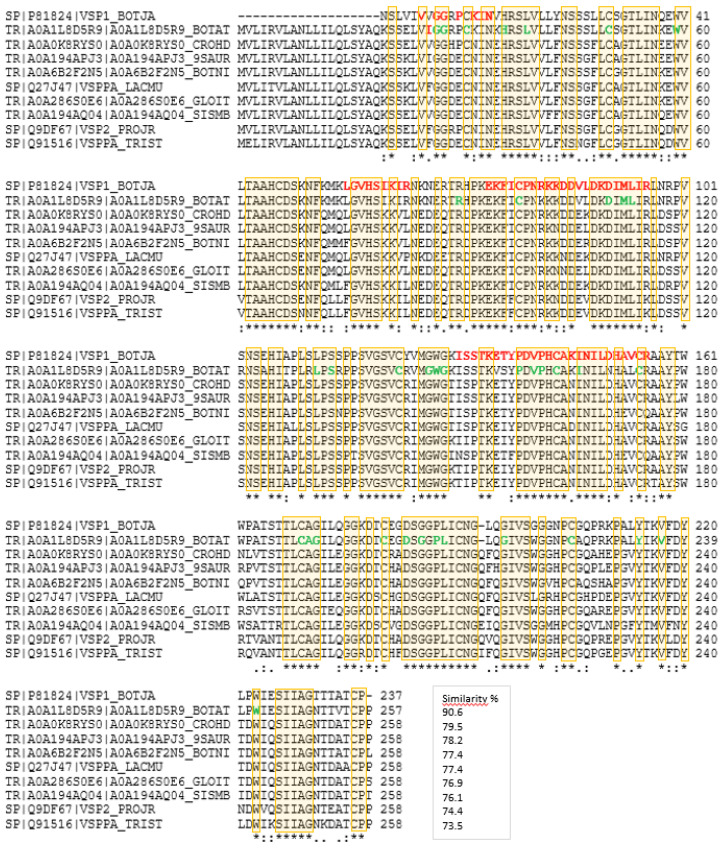
BLAST sequence comparison with the platelet-aggregating enzyme from *B. jararaca* (P81824) using the Uniprot database. Sequences from different pit viper species (Viperidae/Crotalinae: rattlers, moccasins, bushmasters, and lanceheads) were chosen for illustration down to a 73% sequence similarity. Areas marked in orange and with a star are identical. Red/bold AA residues have been confirmed by target MS/MS and Edman sequencing. Green/bold are residues, which are conserved in 996 homologous sequences, as determined with BLAST. Identical AA are indicated by “*”, related AA by “:”.

**Figure 6:**
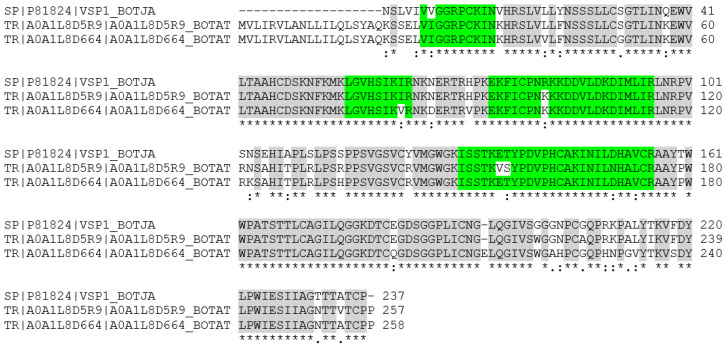
Sequence alignment of the platelet-aggregating enzyme from *B. jararaca* (P81824) and two *B. atrox* proteins sharing the experimentally validated sequence parts of the 31 kDa protein. Identical AA are indicated by “*”, related AA by “:”. Green colour indicates the sequence regions detected, grey are the shared AA among sequences.

Sequence homology analysis using the Uniprot BLAST algorithm for the snake venom serine protease (SVSP) from *B. jararaca* (P81824, Figure 4) provided about 900 similar venom proteins from different viper species with sequence homologies better than 60%. For an illustration, a CLUSTAL O(1.2.4) multiple sequence alignment was performed with a choice of proteins from rattlers, moccasins, bushmasters, and lanceheads, all from the family Viperidae and the subfamily Crotalinae, which were 73–91% homologous (Figure 5). This comparison assists in further sequence elucidation because it shows highly conserved areas. The tryptic peptides, which were confirmed by the target MS/MS, not surprisingly, were located in such homologous regions. All cysteine residues are conserved across these species so that the Edman result for the N-terminus can be further corrected to give the sequence VIGG [R/L] PCKIN. The decision between R_5_ and L_5_ also shifts in the direction of the former because no leucine or isoleucine residue was ever indicated in the other viper proteins at that position. In fact, the sequence alignment analysis detected two proteins from *B. atrox* with 76.7% sequence identity (A0A1L8D5R9 and A0A1L8D664), which matched the other verified sequence areas in the protein, and they had Arg in position 5 (Figure 6). Aspartic acid is often seen in that position also, but neither the presence of Arg nor Asp could be confirmed by the target MS/MS, possibly because the tryptic cleavage sites differ. The two *B. atrox* proteins share large parts of their sequence and seem to be the best candidates for further sequence deduction. All three proteins (P81824, A0A1L8D5R9, and A0A1L8D664) have a theoretical p*I* around 9 (9.08, 9.71, and 9.41; calculated with the Expasy pI/MW tool)) and molecular weights of about 28 kDa (25.741, 28.343, and 28.264 Da), confirming the 2D-PAGE result and providing further evidence for the validity of the identification hypothesis.

## 3. Experimental

### 3.1. Venom Preparation and Protein Isolation

The extracellular part of the human TF was cloned and expressed in *Escherichia coli*. Following the purification, the protein was coupled to CNBr-activated Sepharose 4B (Cytiva) for use as bait. Lyophilized crude *B. moojeni* venom (purchased from Latoxan, Portes-les-Valence, France) was dissolved (10 mg/mL) in 50 mM Tris HCl (pH 8), 75 mM NaCl, and 1 mM EDTA and supplemented with the protease inhibitors PMSF, pepstatin, aprotinin, and leupeptin. The immobilized TF was incubated with the crude *B. moojeni* venom solution on a rotator at 4 °C for 10 min. After washing with the same buffer, the captured proteins were eluted by increasing the NaCl content to 575 mM. The eluate was adjusted to 50 mM NaCl and a 50 mM NaPi buffer (pH 7.5) and concentrated using a Vivaspin 2 centrifugal concentrator (Cytiva). For the N-terminal sequencing, the 31 kDa protein was run on a 12% SDS-PAGE gel, blotted on a PVDF membrane, and stained by Ponceau S. The Edman degradation was performed by the Proteome Factory (Berlin, Germany). For further purification, the 31 kDa protein (0.3 mg/mL in 50 mM NaCl, a 50 mM Na Pi buffer, and pH 7.5) was subjected to a mini-2D-PAGE (pH 3–10, Supplementary Figure S3). The spot visible at about pH 9–10 was excised.

### 3.2. Primary Structure Elucidation by LC-MS/MS

The gel spots were subjected to reduction, alkylation, and tryptic digestion. The peptides were extracted, dried, and redissolved in 10 µL of 0.1% formic acid containing 5% acetonitrile. For the LC-MS/MS and DIA, a Synapt G2 Si coupled to M-Class nanoUPLC (Waters Corp., Manchester, UK) was employed using C18 µPAC columns (trapping and 50 cm analytical; PharmaFluidics, Ghent, Belgium) with a 30 min gradient (10–60%; solvent system 100% water versus 100% acetonitrile, both containing 0.1% formic acid; 0.5 µL injection volume). First, the data-dependent measurements of the most abundant doubly-charged peptide peaks were performed in order to find the MS/MS spectra suitable for sequence tag elucidation. These tags were tested against the Uniprot and NCBI databases using the Protein Prospector pattern search [20]. The sequence hypothesis was subsequently confirmed by target analyses on the tryptic peptides of SVSP from *B. jararaca* (AAB34465.1/P81824) and DIA using this sequence as the database. The sequence ions were assigned as calculated by MassLynx software. The fragment ion tables for the spectra shown here are available in the Supplementary Materials.

## 4. Conclusions

The identification of unknown proteins continues to be a challenge, despite major advances in MS instrumentation and bioinformatic tools, as well as the increase in the number of sequenced proteins. First and foremost, necessary for reliable analysis is the generation of meaningful MS peptide fragmentation spectra. This includes the presence of signals well above the noise of isotopes and of peaks in the information-rich *m/z* region above the parent ion in the spectra of multiply-charged peptides (for a short tutorial, see [2]). If sufficiently long sequence tags of at least six to eight AAs can be derived from such spectra, it is worth running them against the available protein sequence databases to test for homologous proteins. Shorter sequence stretches tend to be too unspecific. This basic method does not require extensive bioinformatics skills or software; peptide MS/MS spectra, especially of doubly-charged ions, can easily be analysed manually, as long as a clear ion fragment series can be detected. For further analysis, one should be familiar with tools such as a pattern search, Blast, and sequence alignment, which are provided, for instance, on the Uniprot platform.

We demonstrated the procedure with the elucidation of a snake venom protease. The application of the above method led to the comparatively fast assignment of highly homologous sequences from related snake species within a week, allowing the biochemists to refocus their research on this particular protein group. However, the new protein was far from fully and correctly sequenced at this point. As both the Edman and the MS results showed, ambiguities remained, even in the sequences’ parts, which matched the hypothesis. An extensive and detailed residue-for-residue analysis will now be necessary to confirm each AA in its position and elucidate the so far not experimentally described sequence regions. If genome sequencing is not an option, MS can be used in combination with various enzymes [13] and the use of dedicated databases containing only the known homologous proteins, but this work will be tedious in particular because the assignment of the isobaric AA and possible modifications requires considerable extra effort.

With the ongoing work in genomics and transcriptomics and the availability of ever more data in public databases, the need for de novo MS sequencing will decrease. However, sequence information is not deposited for every animal and plant species in the databases, so the combination of MS and Edman sequencing with database search tools will continue to be of importance for the identification of unknown proteins for some time.

## Data Availability

All data are given in the results and supplementary sections.

## Supplementary Materials

The following supporting information can be downloaded at: https://www.mdpi.com/article/10.3390/molecules27154976/s1, Word-File: Supplement; Excel-File: Supplement_DIA.

## Sample Availability

The venom samples are available from Latoxan, Portes-les-Valence, France.
