# Supplementary material for: The Current State-of-the-Art Identification of Unknown Proteins Using Mass Spectrometry Exemplified on De Novo Sequencing of a Venom Protease from Bothrops moojeni
Source: Molecules. 2022 Aug 5;27(15):4976. doi: 10.3390/molecules27154976 (PMC9370501; doi:10.3390/molecules27154976)
Supplement: Supplementary file 1 [file molecules-27-04976-s001.zip › molecules-1816712-SI.pdf]

molecules

Feature Article

The current state-of-the-art of the identification of unknown proteins using mass spectrometry exemplified on *de novo* sequencing of a venom protease from *Bothrops moojeni*

Simone König [1], Wolfgang Obermann [2] and Johannes Eble [2]

[1] IZKF Core Unit Proteomics, Interdisciplinary Center for Clinical Research, University of Münster, Röntgenstr. 21, 48149 Münster, Germany; koenigs@uni-muenster.de (S.K.)
[2] Institute of Physiological Chemistry and Pathobiochemistry, University of Münster, Waldeyer-Str., 48149 Münster, Germany; wolfgang.obermann@uni-muenster.de (W.O.); johannes.eble@uni-muenster.de (J.E.)
* Correspondence: koenigs@uni-muenster.de

Citation: Lastname, F.; Lastname, F.; Lastname, F. Title. *Molecules* **2022**, *27*, x. https://doi.org/10.3390/xxxxx

Academic Editor: Firstname Lastname

Received: date
Accepted: date
Published: date

Publisher's Note: MDPI stays neutral with regard to jurisdictional claims in published maps and institutional affiliations.

Copyright: © 2022 by the authors. Submitted for possible open access publication under the terms and conditions of the Creative Commons Attribution (CC BY) license (https://creativecommons.org/licenses/by/4.0/).

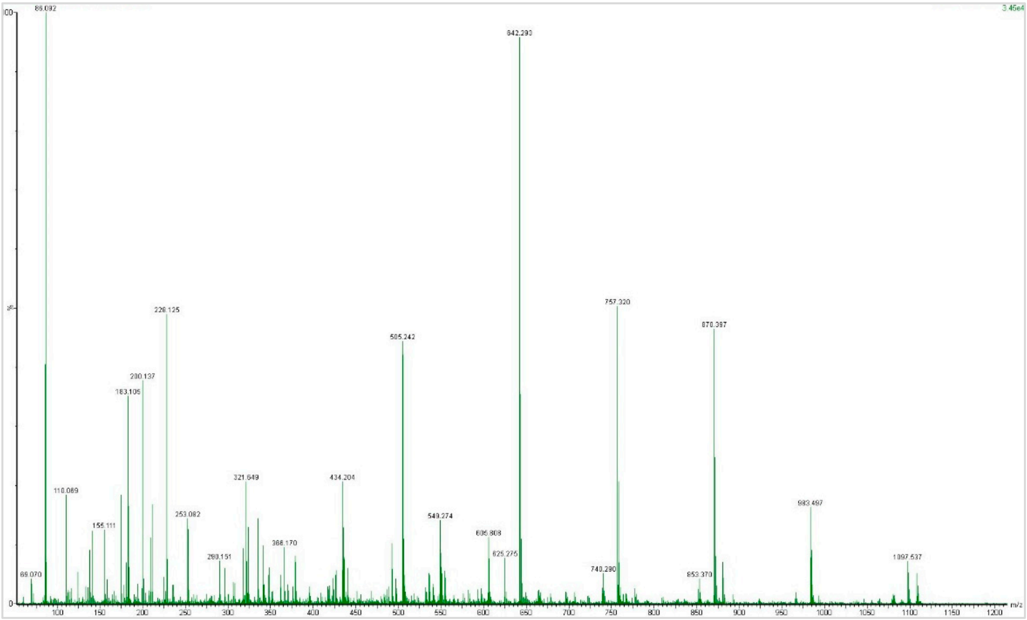

b	114.092	228.135	341.219	454.303	569.330	706.389	777.426	876.494	1036.525	-
i	86.097	87.056	86.097	86.097	88.040	110.072	44.050	72.081	133.044	129.114
b~	96.081	210.124	323.208	436.292	551.319	688.378	759.415	858.484	1018.514	-
b*	97.065	211.108	324.192	437.276	552.303	689.362	760.399	859.468	1019.498	-
	1	2	3	4	5	6	7	8	9	10
	Ile	Asn	Ile	Leu	Asp	His	Ala	Val	CAM	Arg
	10	9	8	7	6	5	4	3	2	1
y"	-	1097.552	983.510	870.425	757.341	642.314	505.256	434.218	335.150	175.119
y~	-	1079.542	965.499	852.415	739.331	624.304	487.245	416.208	317.139	157.109
y*	-	1080.526	966.483	853.399	740.315	625.288	488.229	417.192	318.124	158.093

Figure S1. MS/MS spectrum of the doubly-charged peptide ion (*m/z* 605.79) assigned to peptide 142-155 (AAB34465.1) detected in the tryptic digest of a 31 kDa protein isolated from *B. moojeni* and the calculation of the expected fragment ions for sequence INILDHAVCamR using MassLynx.

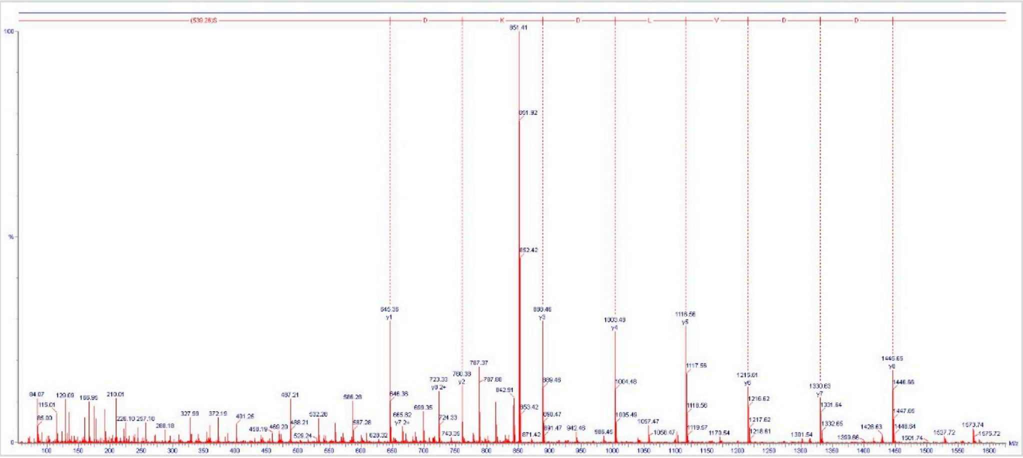

Figure S2. MS/MS spectrum of the doubly-charged peptide ion (*m*/*z* 851.05) detected in the tryptic digest of a 31 kDa protein isolated from *B. moojeni* analyzed by the MassLynx PepSeq program. A seven- amino acid residue tag was suggested.

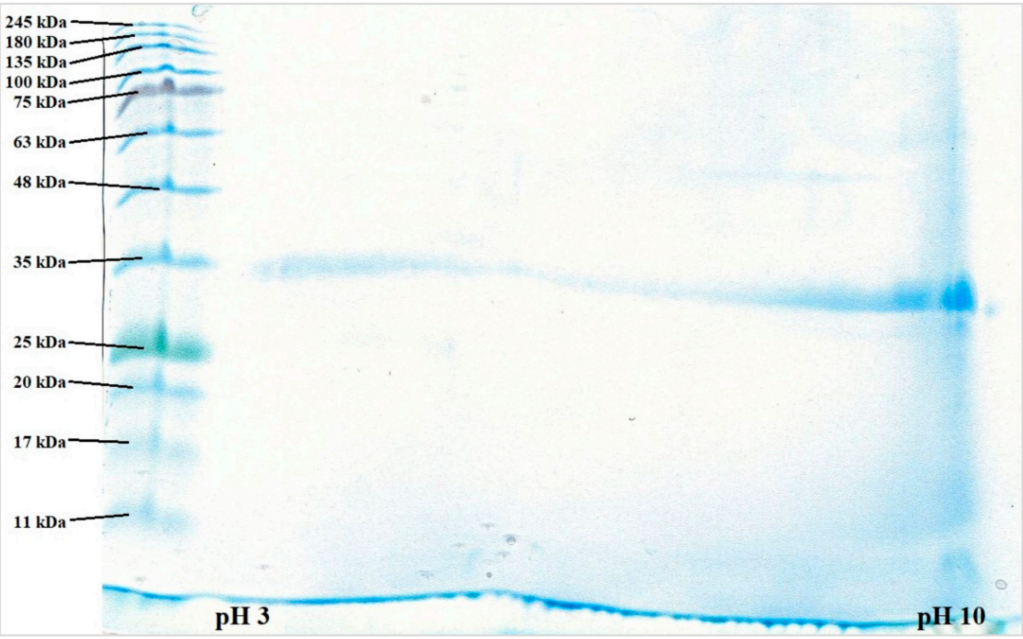

Figure S3. Mini-2D-gel of S/MS spectra of a 31 kDa protein isolated from *B. moojeni*. The protein spot at pH 10 was excised from the gel and subjected to tryptic digestion and MS analysis.

.

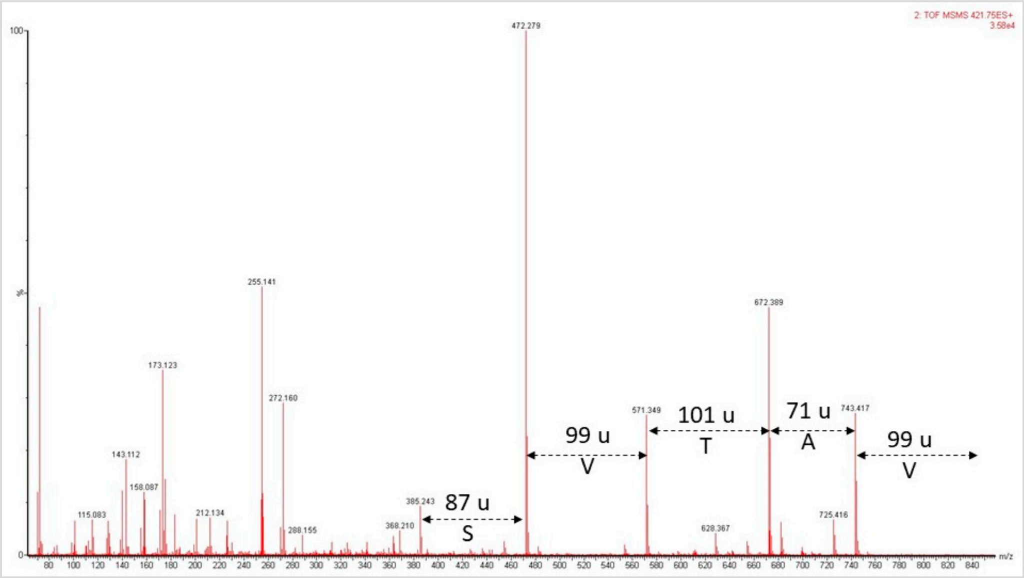

Figure S4. MS/MS spectrum of a doubly-charged ion detected at *m*/*z* 421.75 assigned to sequence VATVSLPR from digestion enzyme trypsin (P00761, TRYP_PIG) used in sample preparation.

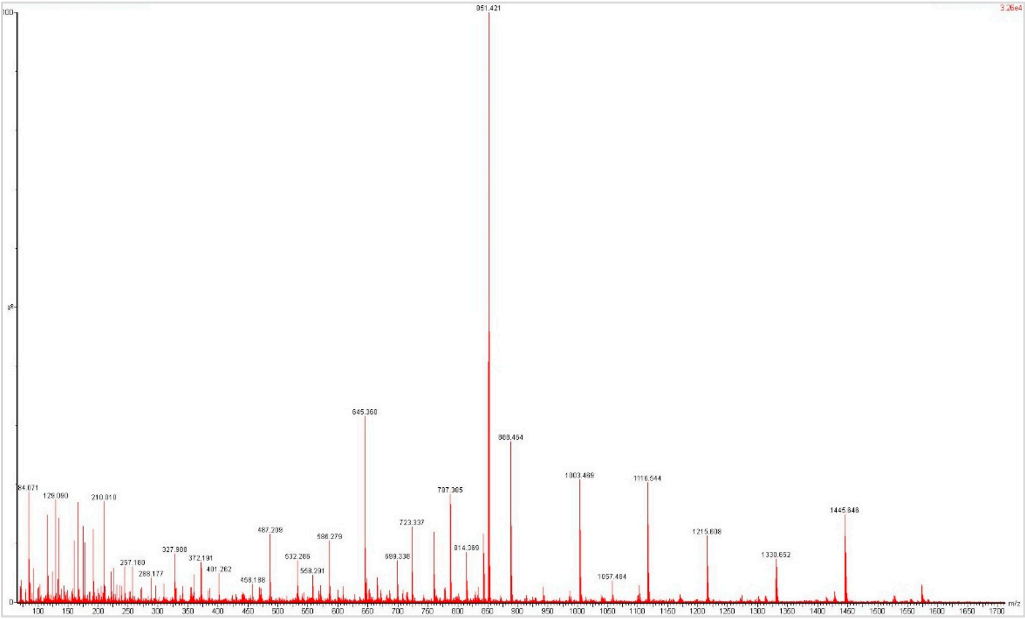

Figure S5. MS/MS spectrum of a doubly-charged peptide ion (*m*/*z* 851.05) detected in the tryptic digest of a 31 kDa protein isolated from *B. moojeni*. See Figure S2 for PepSeq assignment.

b	129.103	257.198	372.225	487.252	586.320	699.404	814.431	942.526	1057.553	1170.637	1301.677	1414.762	1527.846	-
i	101.108	101.108	88.040	88.040	72.081	86.097	88.040	101.108	88.040	86.097	104.053	86.097	86.097	129.114
b~	111.092	239.187	354.214	469.241	568.309	681.394	796.420	924.515	1039.542	1152.626	1283.667	1396.751	1509.835	-
b*	112.076	240.171	355.198	470.225	569.293	682.378	797.404	925.499	1040.526	1153.610	1284.651	1397.735	1510.819	-
	1	2	3	4	5	6	7	8	9	10	11	12	13	14
	Lys	Lys	Asp	Asp	Val	Leu	Asp	Lys	Asp	Ile	Met	Leu	Ile	Arg
	14	13	12	11	10	9	8	7	6	5	4	3	2	1
y''	-	1573.862	1445.767	1330.740	1215.714	1116.645	1003.561	888.534	760.439	645.412	532.328	401.288	288.204	175.119
y~	-	1555.852	1427.757	1312.730	1197.703	1098.634	985.550	870.523	742.429	627.402	514.318	383.277	270.193	157.109
y*	-	1556.836	1428.741	1313.714	1198.687	1099.619	986.534	871.507	743.413	628.386	515.302	384.261	271.177	158.093

Figure S6. Expected ion series for sequence KKDVLDKDIMLIR (AA 78-91, AAB34465.1) as calculated using MassLynx. See Figure S5 for original data.

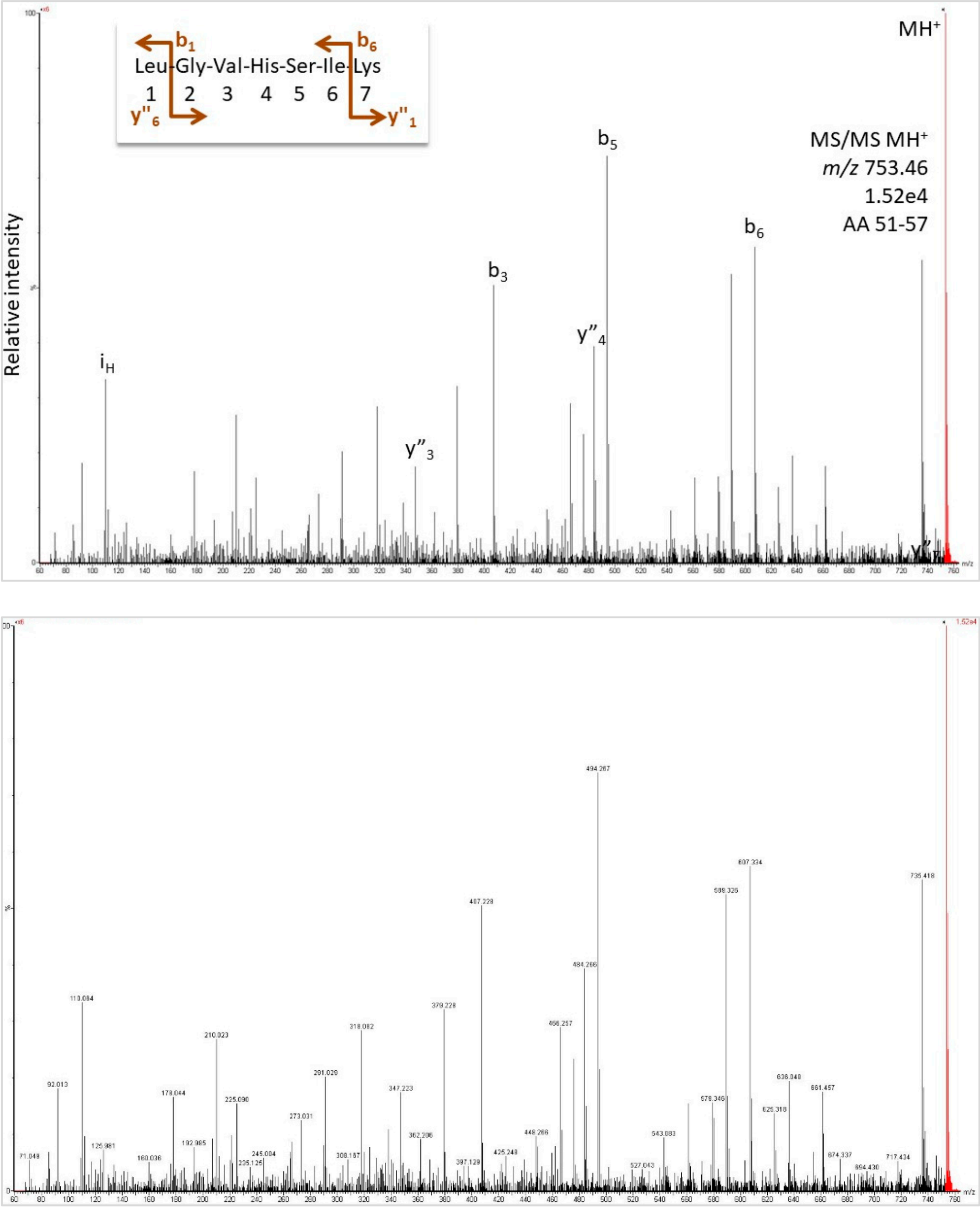

b	114.092	171.113	270.182	407.241	494.273	607.357	-
i	86.097	30.034	72.081	110.072	60.045	86.097	101.108
b~	96.081	153.103	252.171	389.230	476.262	589.346	-
b*	97.065	154.087	253.155	390.214	477.246	590.330	-
	1	2	3	4	5	6	7
	**Leu**	**Gly**	**Val**	**His**	**Ser**	**Ile**	**Lys**
	7	6	5	4	3	2	1
y"	-	640.378	583.357	484.288	347.229	260.197	147.113
y~	-	622.368	565.346	466.278	329.219	242.187	129.103
y*	-	623.352	566.330	467.262	330.203	243.171	130.087

Figure S7. Original and assigned MS/MS spectrum of the singly-charged peptide ion (*m/z* 753.46) of peptide 51-57 (AAB34465.1) detected in the tryptic digest of a 31 kDa protein isolated from *B. moojeni* and the calculation of the expected fragment ions for sequence LGVHSIK using MassLynx.

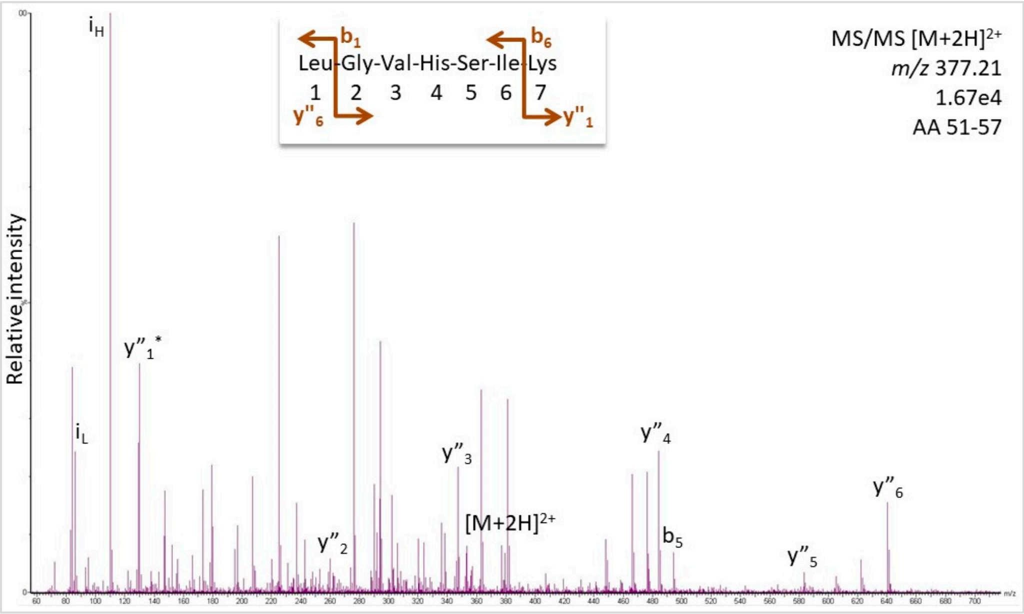

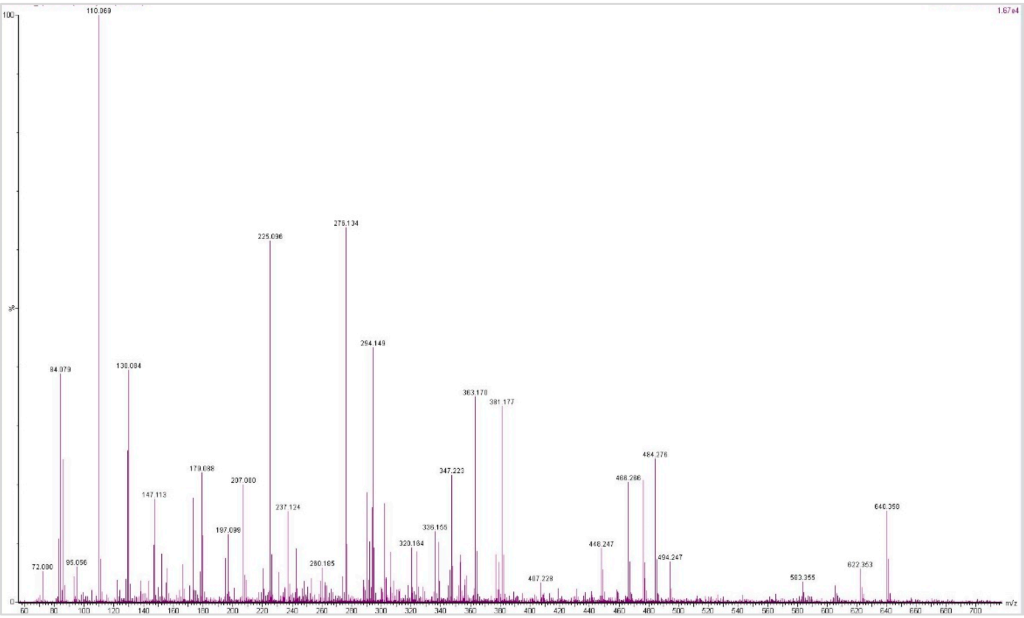

b	114.092	171.113	270.182	407.241	494.273	607.357	-
i	86.097	30.034	72.081	110.072	60.045	86.097	101.108
b~	96.081	153.103	252.171	389.230	476.262	589.346	-
b*	97.065	154.087	253.155	390.214	477.246	590.330	-
	1	2	3	4	5	6	7
	Leu	Gly	Val	His	Ser	Ile	Lys
	7	6	5	4	3	2	1
y"	-	640.378	583.357	484.288	347.229	260.197	147.113
y~	-	622.368	565.346	466.278	329.219	242.187	129.103
y*	-	623.352	566.330	467.262	330.203	243.171	130.087

Figure S8. Original and assigned MS/MS spectrum of the doubly-charged peptide ion (*m/z* 377.21) of peptide 51-57 (AAB34465.1) detected in the tryptic digest of a 31 kDa protein isolated from *B. moojeni* and the calculation of the expected fragment ions for sequence LGVHSIK using MassLynx.

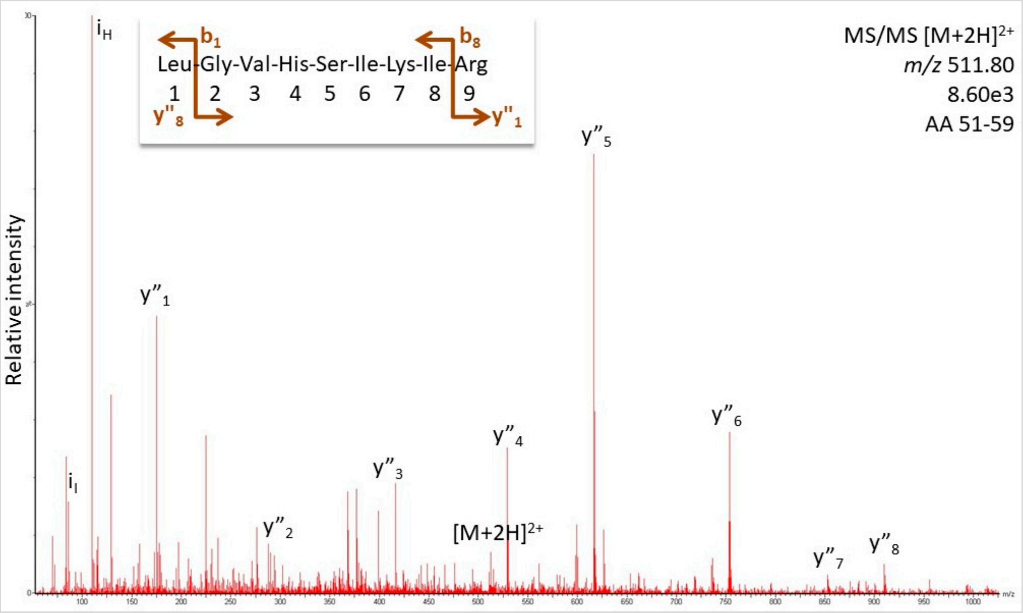

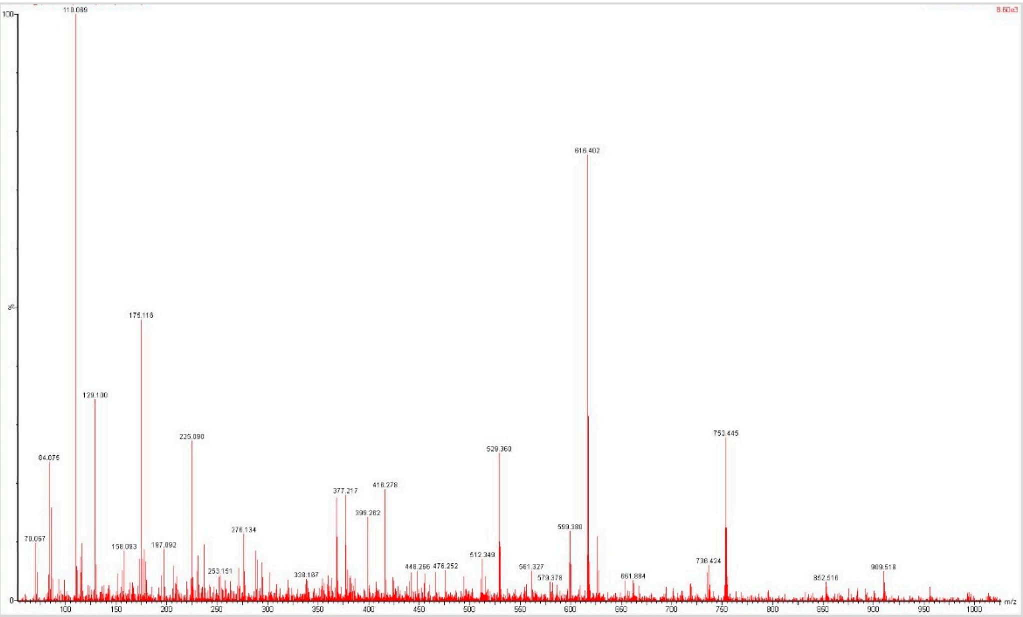

b	114.092	171.113	270.182	407.241	494.273	607.357	735.452	848.536	-
i	86.097	30.034	72.081	110.072	60.045	86.097	101.108	86.097	129.114
b~	96.081	153.103	252.171	389.230	476.262	589.346	717.441	830.525	-
b*	97.065	154.087	253.155	390.214	477.246	590.330	718.425	831.509	-
	1	2	3	4	5	6	7	8	9
	Leu	Gly	Val	His	Ser	Ile	Lys	Ile	Arg
	9	8	7	6	5	4	3	2	1
y"	-	909.563	852.542	753.473	616.415	529.383	416.298	288.204	175.119
y~	-	891.553	834.531	735.463	598.404	511.372	398.288	270.193	157.109
y*	-	892.537	835.515	736.447	599.388	512.356	399.272	271.177	158.093

Figure S9. Original and assigned MS/MS spectrum of the doubly-charged peptide ion (*m*/*z* 511.80) of peptide 51-59 (AAB34465.1) detected in the tryptic digest of a 31 kDa protein isolated from *B. moojeni* and the calculation of the expected fragment ions for sequence LGVHSIKIR using MassLynx.

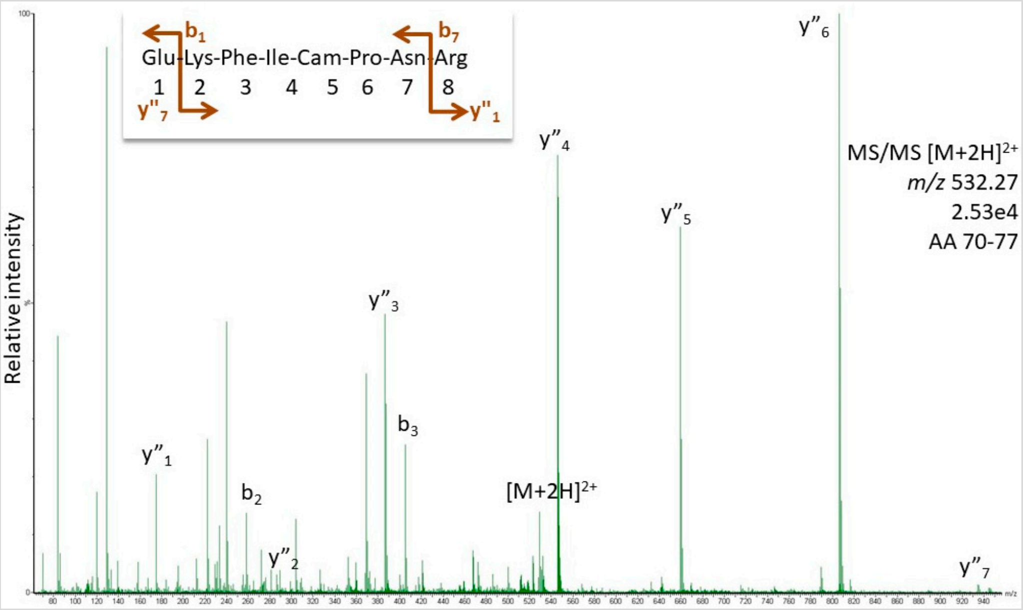

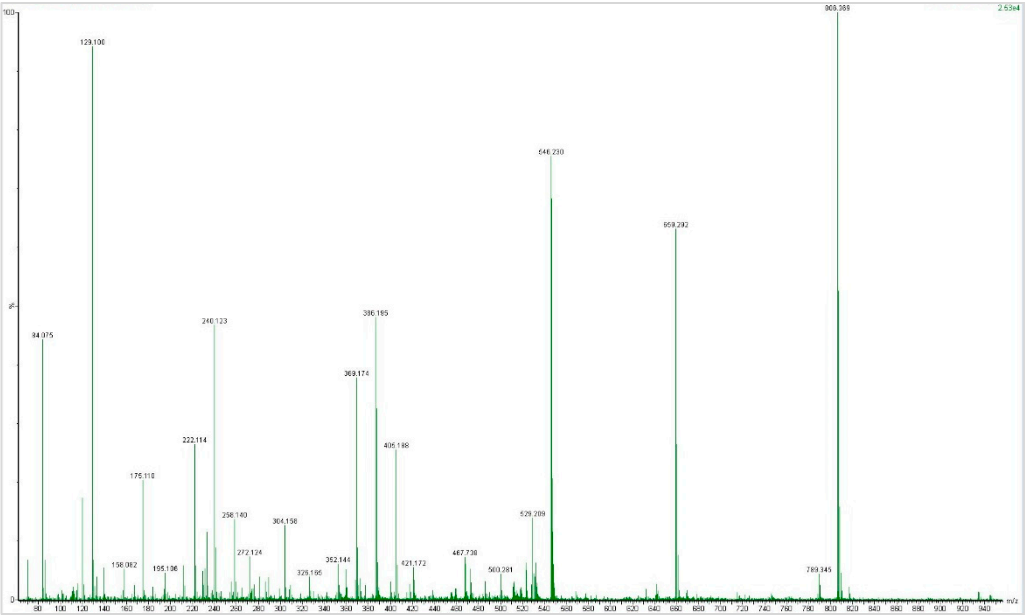

	1	2	3	4	5	6	7	8
**b**	130.050	258.145	405.214	518.298	678.328	775.381	889.424	-
**i**	102.056	101.108	120.081	86.097	133.044	70.066	87.056	129.114
**b~**	112.040	240.135	387.203	500.287	660.318	757.371	871.414	-
**b***	113.024	241.119	388.187	501.271	661.302	758.355	872.398	-
	**Glu**	**Lys**	**Phe**	**Ile**	**CAM**	**Pro**	**Asn**	**Arg**
	8	7	6	5	4	3	2	1
**y"**	-	934.493	806.398	659.330	546.246	386.215	289.162	175.119
**y~**	-	916.483	788.388	641.319	528.235	368.205	271.152	157.109
**y***	-	917.467	789.372	642.303	529.219	369.189	272.136	158.093

Figure S10. Original and assigned MS/MS spectrum of the doubly-charged peptide ion (*m/z* 532.27) of peptide 70-77 (AAB34465.1) detected in the tryptic digest of a 31 kDa protein isolated from *B. moojeni* and the calculation of the expected fragment ions for sequence EKFICamPNR using MassLynx.

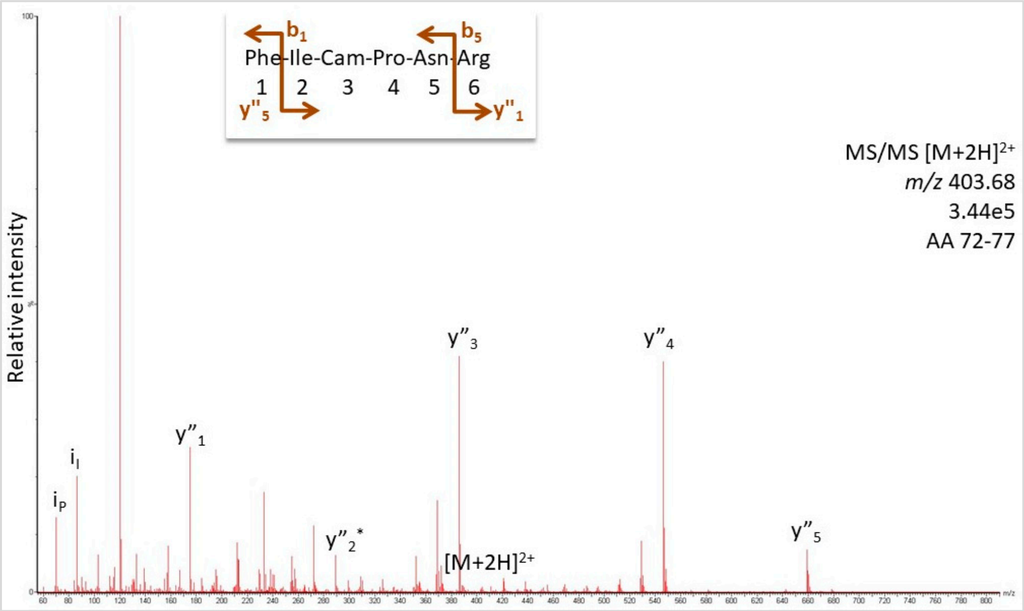

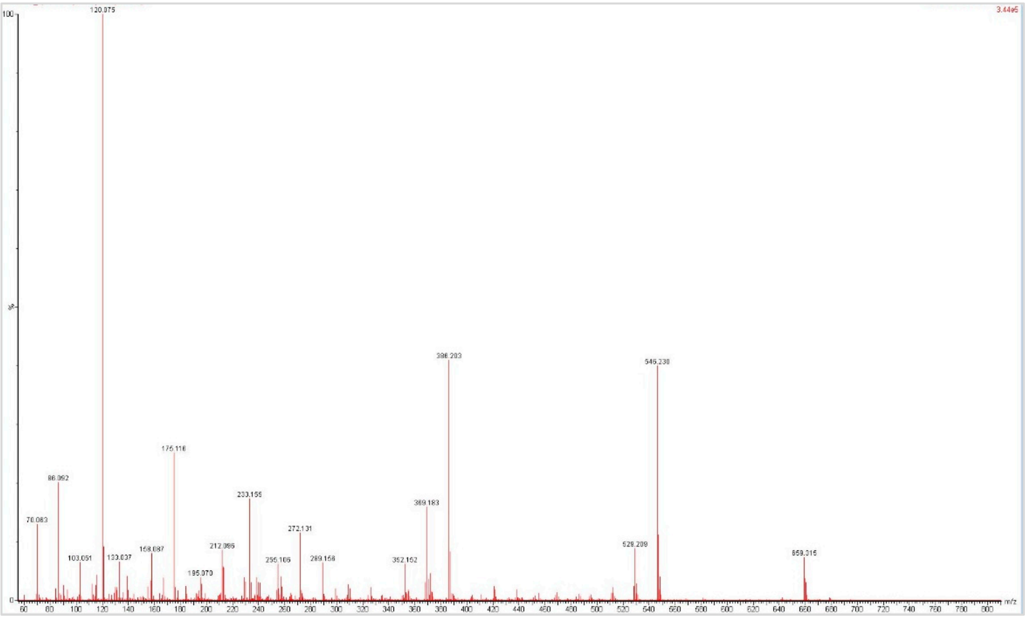

b	148.076	261.160	421.191	518.244	632.287	-
i	120.081	86.097	133.044	70.066	87.056	129.114
b~	130.066	243.150	403.180	500.233	614.276	-
b*	131.050	244.134	404.164	501.217	615.260	-
	1	2	3	4	5	6
	Phe	Ile	CAM	Pro	Asn	Arg
	6	5	4	3	2	1
y"	-	659.330	546.246	386.215	289.162	175.119
y~	-	641.319	528.235	368.205	271.152	157.109
y*	-	642.303	529.219	369.189	272.136	158.093

Figure S11. Original and assigned MS/MS spectrum of the doubly-charged peptide ion (*m*/*z* 403.68) of peptide 72-77 (AAB34465.1) detected in the tryptic digest of a 31 kDa protein isolated from *B. moojeni* and the calculation of the expected fragment ions for sequence FICamPNR using MassLynx.

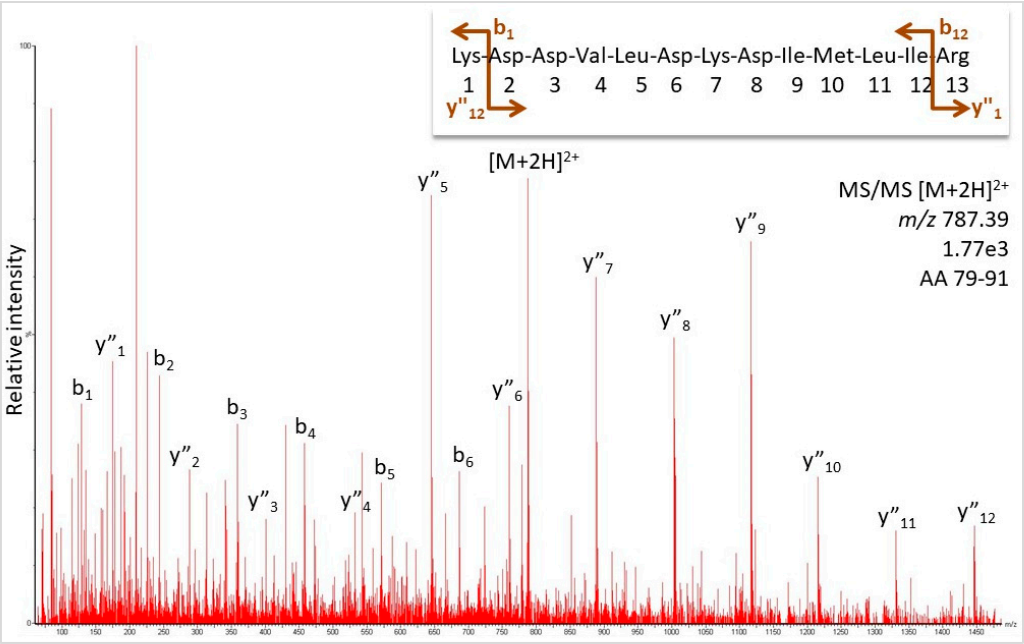

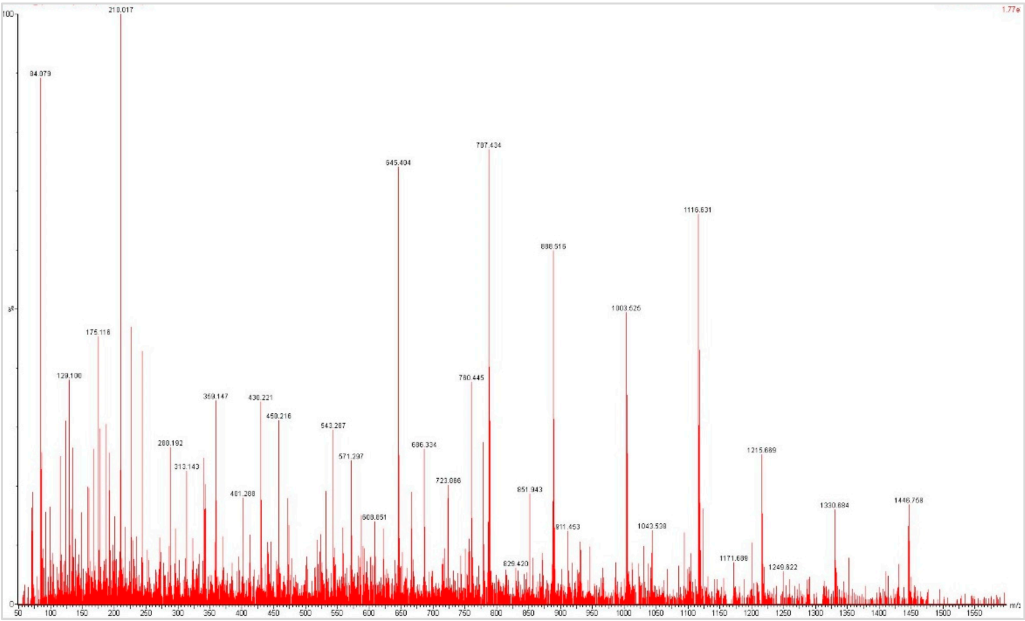

b	129.103	244.130	359.157	458.225	571.309	686.336	814.431	929.458	1042.542	1173.583	1286.667	1399.751	-
i	101.108	88.040	88.040	72.081	86.097	88.040	101.108	88.040	86.097	104.053	86.097	86.097	129.114
b~	111.092	226.119	341.146	440.215	553.299	668.326	796.420	911.447	1024.531	1155.572	1268.656	1381.740	-
b*	112.076	227.103	342.130	441.199	554.283	669.310	797.404	912.431	1025.516	1156.556	1269.640	1382.724	-
	1	2	3	4	5	6	7	8	9	10	11	12	13
	Lys	Asp	Asp	Val	Leu	Asp	Lys	Asp	Ile	Met	Leu	Ile	Arg
	13	12	11	10	9	8	7	6	5	4	3	2	1
y"	-	1445.767	1330.740	1215.714	1116.645	1003.561	888.534	760.439	645.412	532.328	401.288	288.204	175.119
y~	-	1427.757	1312.730	1197.703	1098.634	985.550	870.523	742.429	627.402	514.318	383.277	270.193	157.109
y*	-	1428.741	1313.714	1198.687	1099.619	986.534	871.507	743.413	628.386	515.302	384.261	271.177	158.093

Figure S12. Original and assigned MS/MS spectrum of a doubly-charged peptide ion (*m/z* 787.39) detected in the tryptic digest of a 31 kDa protein isolated from *B. moojeni* and the calculation of the expected fragment ions for sequence KDVLDKDIMLIR (AA 79-91, AAB34465.1) using MassLynx.

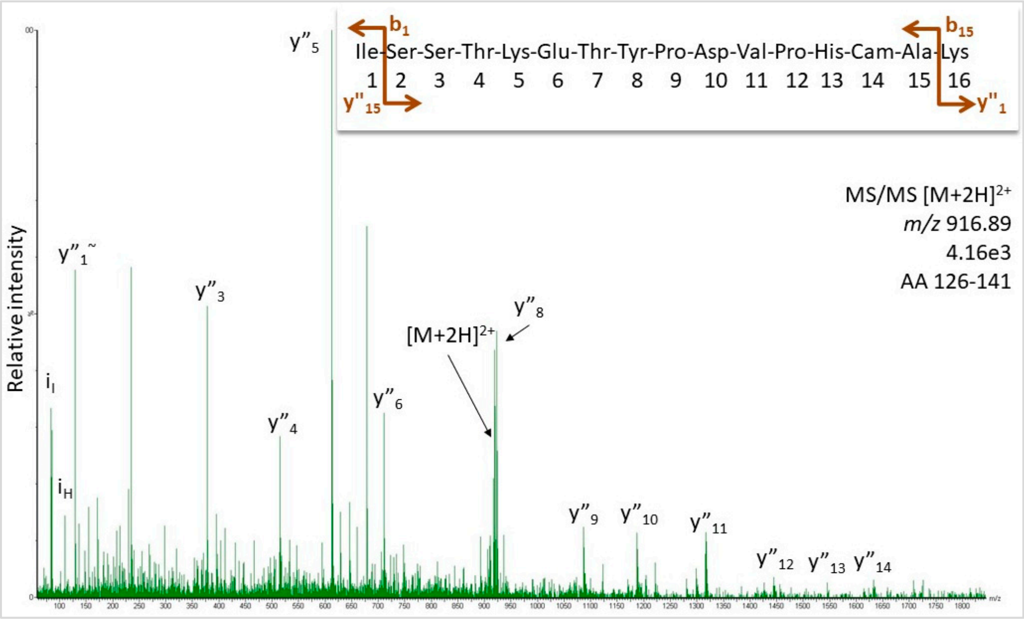

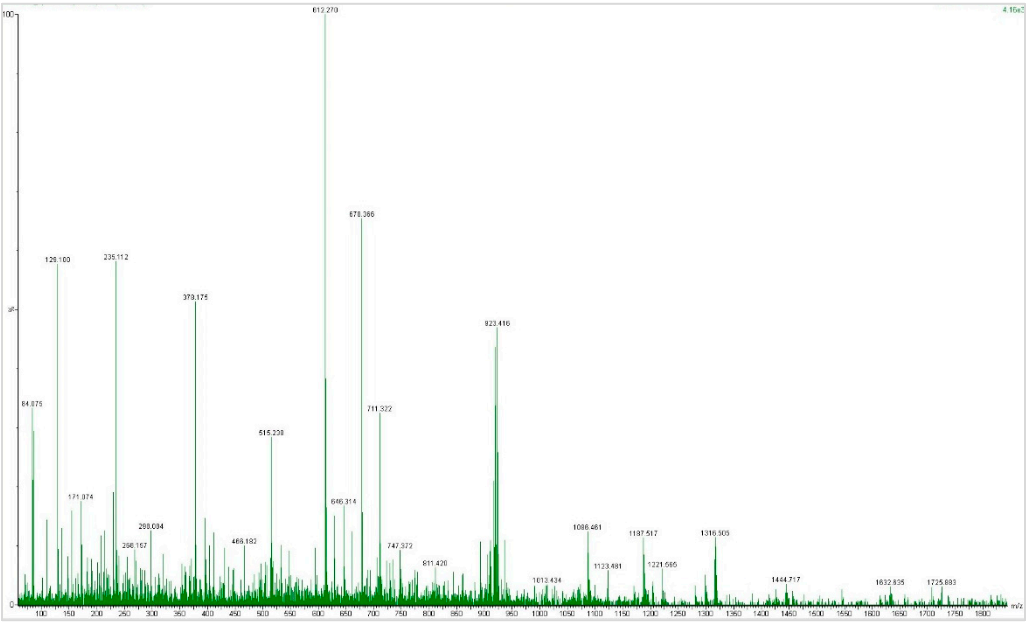

	1	2	3	4	5	6	7	8	9	10	11	12	13	14	15	16
b	114.092	201.124	288.156	389.204	517.299	646.341	747.389	910.452	1007.505	1122.532	1221.600	1318.653	1455.712	1615.743	1686.780	-
i	86.097	60.045	60.045	74.061	101.108	102.056	74.061	136.076	70.066	88.040	72.081	70.066	110.072	133.044	44.050	101.108
b~	96.081	183.113	270.145	371.193	499.288	628.331	729.378	892.442	989.494	1104.521	1203.590	1300.642	1437.701	1597.732	1668.769	-
b*	97.065	184.097	271.129	372.177	500.272	629.315	730.362	893.426	990.478	1105.505	1204.574	1301.627	1438.685	1598.716	1669.753	-
	Ile	Ser	Ser	Thr	Lys	Glu	Thr	Tyr	Pro	Asp	Val	Pro	His	CAM	Ala	Lys
	16	15	14	13	12	11	10	9	8	7	6	5	4	3	2	1
y"	-	1719.801	1632.769	1545.737	1444.689	1316.594	1187.552	1086.504	923.441	826.388	711.361	612.293	515.240	378.181	218.150	147.113
y~	-	1701.791	1614.758	1527.726	1426.679	1298.584	1169.541	1068.494	905.430	808.378	693.351	594.282	497.229	360.170	200.140	129.103
y*	-	1702.775	1615.743	1528.711	1427.663	1299.568	1170.525	1069.478	906.414	809.362	694.335	595.266	498.213	361.154	201.124	130.087

Figure S13. Original and assigned MS/MS spectrum of a doubly-charged peptide ion (*m*/*z* 919.89) detected in the tryptic digest of a 31 kDa protein isolated from *B. moojeni* and the calculation of the expected fragment ions for sequence ISSTKETYPDVPHCamAK (AA 126-141, AAB34465.1) using MassLynx.

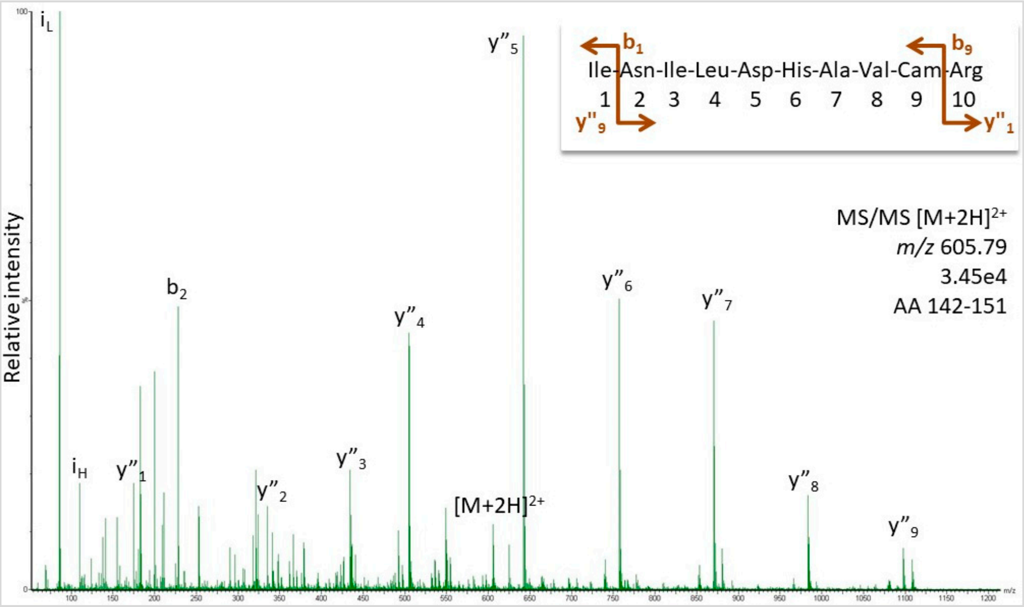

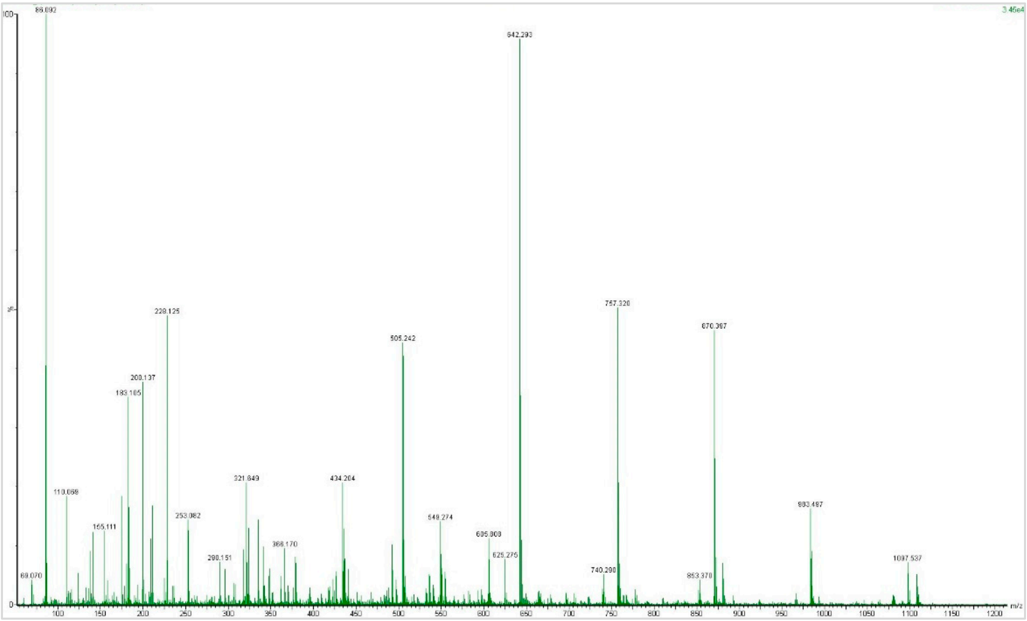

b	114.092	228.135	341.219	454.303	569.330	706.389	777.426	876.494	1036.525	-
i	86.097	87.056	86.097	86.097	88.040	110.072	44.050	72.081	133.044	129.114
b~	96.081	210.124	323.208	436.292	551.319	688.378	759.415	858.484	1018.514	-
b*	97.065	211.108	324.192	437.276	552.303	689.362	760.399	859.468	1019.498	-
	1	2	3	4	5	6	7	8	9	10
	Ile	Asn	Ile	Leu	Asp	His	Ala	Val	CAM	Arg
	10	9	8	7	6	5	4	3	2	1
y"	-	1097.552	983.510	870.425	757.341	642.314	505.256	434.218	335.150	175.119
y~	-	1079.542	965.499	852.415	739.331	624.304	487.245	416.208	317.139	157.109
y*	-	1080.526	966.483	853.399	740.315	625.288	488.229	417.192	318.124	158.093

Figure S14. Original and assigned MS/MS spectrum of a doubly-charged peptide ion (*m/z* 605.79) detected in the tryptic digest of a 31 kDa protein isolated from *B. moojeni* and the calculation of the expected fragment ions for sequence INILDHAVCamR (AA 142-151, AAB34465.1) using MassLynx.
